# Seismic and mineralogical evidence for an iron-rich mega–ultralow-velocity zone beneath Hawai’i

**DOI:** 10.1126/sciadv.adz1962

**Published:** 2026-01-28

**Authors:** Doyeon Kim, Jung-Hun Song, Vasilije V. Dobrosavljevic, Vedran Lekić

**Affiliations:** ^1^ Department of Earth Science and Engineering, Imperial College London, London, UK.; ^2^ The Research Institute of Basic Sciences, Seoul National University, Seoul, South Korea.; ^3^ Earth & Planets Laboratory, Carnegie Institution for Science, Washington, DC, USA.; ^4^ Department of Geology, University of Maryland, College Park, MD, USA.

## Abstract

Mantle plumes beneath major oceanic hot spots appear to be rooted in unusually large structures near the core-mantle boundary, which have markedly reduced seismic wave speeds. The origin of these large ultralow-velocity zones (ULVZs), referred to as mega-ULVZs, remains uncertain partly because of lack of constraints on the relative reduction in shear versus compressional wave speeds (*R*_S/P_). This ratio can give clues into the compositional makeup of the mega-ULVZs. Through joint seismic analysis of core-diffracted *P* and *S* waves beneath Hawai’i, we constrain the *R*_S/P_ of its mega-ULVZ to 1 to 1.3. Mineralogical modeling reveals that iron enrichment via solid iron-rich magnesiowüstite [(Mg,Fe)O] matches this seismic constraint, independent of modeled ULVZ thickness. Enrichment of metallic iron-rich magnesiowüstite likely enhances the thermal conductivity of mega-ULVZs and provides a mechanism to drive localized plume upwelling. Higher reported *R*_S/P_ values for smaller ULVZs near subduction zones may therefore indicate different processes at play controlling ULVZ formation across the diverse core-mantle boundary landscape.

## Teaser

Hawai’i’s volcanic roots trace back to dense, iron-rich relics deep within Earth’s mantle.

## INTRODUCTION

Earth’s lower mantle hosts a panoply of anomalous structures across various scales. Among these are two continent-sized structures known as large low-velocity provinces (LLVPs) (*1*), consistently imaged in three-dimensional (3D) models of Earth’s interior (*2*). Smaller features, such as ultralow-velocity zones (ULVZs) (*3*) and relatively larger “mega-ULVZs” (*4*), remain largely beyond the resolution of current global tomographic approaches. However, they have been investigated using waveform modeling of seismic phases that interact with core-mantle boundary (CMB) heterogeneities through scattering or multipathing (*5*, *6*). Recent studies suggest that these structures may harbor primordial geochemical reservoirs (*7*–*9*), linking them closely to fundamental geological processes, including core-mantle interactions facilitated by mantle plumes.

The origin and dynamics of these CMB structures remain highly uncertain. Mantle plumes beneath major oceanic hot spots, such as those under Hawai’i (*6*), Iceland (*10*), Galapagos (*11*), Marquesas (*12*), Samoa (*4*), and Pitcairn (*13*), appear to originate from unusually extensive mega-ULVZs, characterized by size, morphology, and notably reduced shear wave speed ($V_{S}$) compared to typical ULVZs found throughout the CMB (*3*). Here, we use “mega” in a relative sense to describe ULVZs at the extreme end of the spectrum, characterized by lateral extents in excess of several hundred kilometers. Geodynamical studies of the interplay between the distribution of mega-ULVZs and overall mantle circulation have sought to explain their morphology (*14*–*16*). Some plume-derived lavas from these hot spots exhibit elevated ^3^He/^4^He ratios anticorrelated with μ^182^W anomalies, implying a connection to mega-ULVZs as potential reservoirs of primitive geochemical signatures dating to Earth’s deepest past (*8*). Such isotopic anomalies could result from mantle processes or core-mantle isotopic equilibration facilitated by partial melting in mega-ULVZs (*17*–*19*).

Key insights into the seismic characteristics of mega-ULVZs derive from postcursors arising from *S* waves diffracting along the CMB (*S*_diff_) (*6*) or waves traversing the core and partly diffracting along the CMB (*SPdKS*) (*4*). These seismic phases provide extensive spatial coverage at the base of the mantle, and analyses of their timing and amplitude help constrain relative dimensions and $V_{S}$ reductions ($\delta V_{S}$) to the surrounding mantle materials required for mega-ULVZs. Core-reflected *S* waves (*ScS*), typically analyzed at shorter periods than *S*_diff_ or *SPdKS*, can further capture the intricate structure of these zones (*20*, *21*). Although many studies have targeted individual mega-ULVZs, systematic investigations that search across broader geographic regions are only recently emerging (*12*, *22*). In addition, deciphering the composition and origin of mega-ULVZs necessitates constraints on compressional wave speed ($V_{P}$) and density—information that *S*-wave analysis alone cannot resolve.

The *P*-wave counterparts of *S*_diff_ (*P*_diff_) offer a solution, although only a few studies have used them for ULVZ analysis (*23*–*25*). The limited use of *P*_diff_ arrivals in studying CMB structures stems from several factors: (i) typically weaker signal strength relative to *S*_diff_, (ii) reduced P-to-P scattering because of ULVZs exhibiting greater $V_{S}$ than $V_{P}$ drops, (iii) lower signal-to-noise ratios in shorter-period *P* waves, and (iv) contamination of the *P*-wave coda by waves that have converted to *S* wave because of receiver-side structures. Here, we present systematic observations of *P*_diff_ postcursors from the mega-ULVZ beneath Hawai’i. By analyzing waveform similarities, we trace *P*_diff_ postcursor patterns geographically and identify their moveout, enabling measurements of amplitude decay analogous to those used in the recent *S*_diff_ postcursor analysis (*12*). A joint analysis of *P*- and *S*-wave datasets, coupled with 3D wavefield simulations, enables estimates of relative reduction in $V_{S}$ versus $V_{P}$ (i.e., $\mathrm{\delta ln}V_{S}$/$\mathrm{\delta ln}V_{P}$, henceforth *R*_S/P_) for the Hawai’ian mega-ULVZ. Leveraging this new estimate, we conduct mineral physics modeling to explore its implications for the composition and physical state of the Hawai’ian mega-ULVZ.

## RESULTS

**Fig. 1.**
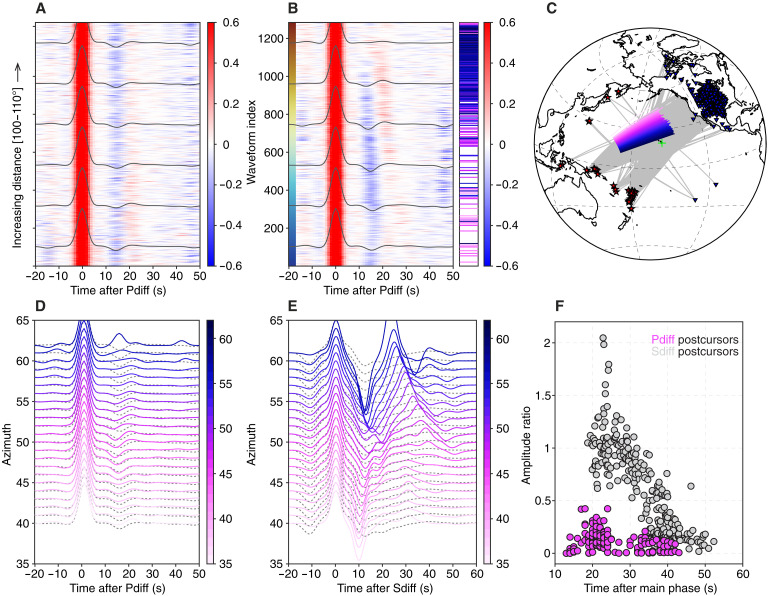
*P*_diff_ waveform data in the Hawai’i region. (**A**) Vertical component of source deconvolved *P*_diff_ displacement waveforms from *M*_w_ > 6.5 earthquakes at epicentral distances between 100° and 110°. The red-blue color scale shows normalized amplitude relative to the main *P*_diff_ arrival. Events with hypocenter depths greater than 200 km are included in the analysis. (**B**) Sequenced *P*_diff_ waveforms, with the left-hand color bar indicating the sequence index, which is referenced in Fig. 2 to illustrate the geographic distribution of postcursors. Horizontal bars on the right side indicate waveforms from a representative event, with colors denoting azimuths corresponding to (C) to (E). (**C**) Map showing earthquake epicenters (red) and seismic stations (blue) used in this study. Highlighted paths correspond to azimuth-sorted recordings from the example event shown in (D) and (E). (**D**) *P*_diff_ and (**E**) *S*_diff_ waveforms from *M*_w_ 6.6 earthquake on 2010 March 20 (New Ireland Region) sorted by azimuth. Dashed waveforms represent average waveforms calculated from the first half of the sequenced waveforms in (B) and from ref. (*12*), which do not exhibit clear postcursor arrivals. (**F**) Amplitude ratios and delay times of post-*P*_diff_ and post-*S*_diff_ arrivals shown in (D) and (E).

We analyzed vertical-component waveforms from earthquakes with moment magnitude (*M*_w_) > 6.5 recorded by broadband seismic stations at epicentral distances between 100° and 110° from 1990 to 2021 (Materials and Methods). To avoid contamination by body wave signals produced by the shallow crustal structure including depth phases, we exclusively take events with a hypocenter depth greater than 200 km. The instrument response was removed from the raw data, and the resulting displacement seismograms were bandpass filtered between 5 and 100 s using a Butterworth filter. The waveforms were then windowed around the *P*_diff_ phase, starting 30 s before and ending 70 s after the predicted *P*_diff_ arrival time, as determined using the Preliminary Reference Earth Model (PREM) (Fig. 1A) (*26*). To minimize the effects of source radiation pattern variations, the *P*_diff_ waveforms were deconvolved using synthetic waveforms computed for PREM (fig. S1) (*27*) and centroid moment tensors from www.globalcmt.org. Here, we only focus on characterizing the CMB region beneath Hawai’i by selecting waveforms with turning points within 20° of the Hawai’ian hot spot.

For comparison, we included tangential-component waveforms of *S*_diff_ from previous work (*12*). These waveforms were processed in the same manner as the *P*_diff_ data, with the exception of applying a bandpass filter between 15 and 100 s, which results in comparable wavelengths for both *P* and *S* waves at the CMB. Typically, *S*_diff_ postcursors associated with a mega-ULVZ with $\delta V_{S}$ exceeding 10% are identified by sorting waveforms by azimuth for each earthquake event individually (*6*). To extend this analysis beyond single-event explorations and cover larger geographic regions that may contain multiple mega-ULVZs, waveforms can instead be ordered on the basis of their intrinsic similarity using the Sequencer (Materials and Methods) (*28*). The Sequencer contextualizes full waveforms by sorting them into coherent patterns without relying on predefined models of earthquakes or mantle structure (*12*). In our *P*_diff_ dataset, applying the Sequencer successfully revealed distinct long period postcursor signals that had not been previously recognized, as shown in Fig. 1B. These signals deviate clearly from the average *P*_diff_ waveform and can be discerned on recordings from individual earthquakes, as shown for the 2010 March 20 New Ireland event in Fig. 1 (C and D). The failure to detect long-period *P*_diff_ postcursors until now likely reflects both their much-smaller amplitudes and the lack of systematic analysis. Even when waveforms are sorted by azimuth alongside clearly visible *S*_diff_ postcursors, the long-period *P*_diff_ postcursors remain difficult to discern (Fig. 1E). Sequenced *P*_diff_ waveforms exhibit postcursor delays of ~10 to 30 s, with amplitudes typically less than 0.3 of the main *P*_diff_ arrival (Materials and Methods) (Fig. 1F). By contrast, *S*_diff_ postcursors show larger delay times (25 to 45 s) and amplitudes approaching or even exceeding that of the main arrival (*12*).

**Fig. 2.**
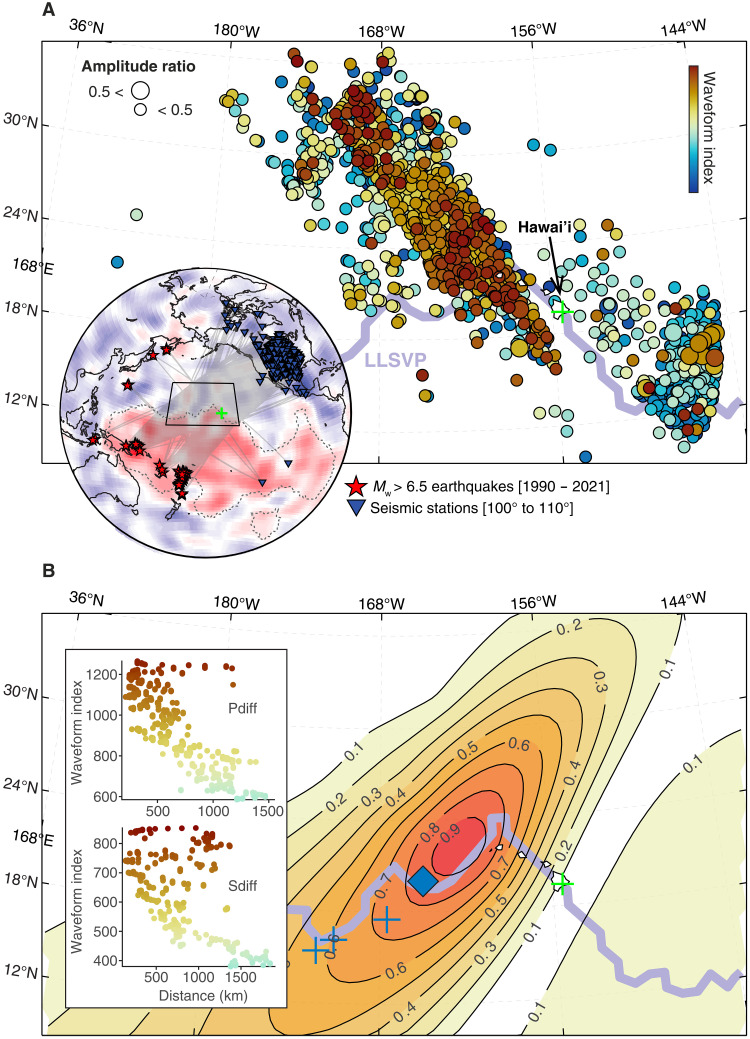
Spatial distribution of the *P*_diff_ postcursors in Hawai’i. (**A**) The midpoints from the diffracting paths are sorted by the sequencing index shown in (B), with symbol size indicating amplitude measurements. The sequence exhibits a consistent geographic pattern, previously identified in the *S*_diff_ postcursors of ref. (*12*), suggesting a common structural source at the CMB. *P*_diff_ amplitudes are well below 0.5, contrasting with the *S*_diff_ analysis [e.g., (F)]. The inset map shows the locations of earthquakes (red) and seismic stations (blue) used in the study, along with the geographic extent of the Pacific LLVP marked by the transition from red to blue regions (*2*). (**B**) Contour plot of the correlation between the sequenced index and the path-midpoint distance associated with each potential location of the mega-ULVZ for combined *P*_diff_ (A) and *S*_diff_ datasets (fig. S2). Blue crosses and diamond symbols indicate previously reported mega-ULVZ locations near Hawai’i using *S*_diff_ and *ScS* waves, respectively. The inset illustrates the relationship between the sequencing index and the midpoint distance at the location exhibiting the highest correlation value.

To visualize the spatial distribution of detected *P*_diff_ postcursors, waveforms were geographically assigned to midpoints of their diffraction paths along the CMB (Fig. 2). We observed a systematic spatial pattern northwest of Hawai’i, characterized by earlier postcursor arrivals surrounding later-arriving signals. This geographic pattern closely matches that reported for *S*_diff_ postcursors (*12*), albeit with somewhat greater variability. Postcursors predominantly appear northwest of Hawai’i, while waveforms without clear postcursors tended to concentrate to the southeast (fig. S2). Furthermore, the sequenced index appears to be correlated with midpoint distance from the location of the previously modeled mega-ULVZ near Hawai’i (brown colors in Fig. 2A). Therefore, for a grid of putative mega-ULVZ locations, we compute the distances to each path midpoint and the correlation coefficient between those distances and the sequenced indices of *P*_diff_ and *S*_diff_ waveforms (fig. S3). For these computations, only data exhibiting clear postcursors (the latter half of the indices) were included, while waveforms without postcursor signals were excluded. The product of the two correlation coefficients is displayed as a contour plot in Fig. 2B and shows a ridge of high sequence-distance correlation to the northwest of Hawai’i, elongated in the direction of the preponderance of the diffracted paths (inset, Fig. 2A). Crossing-path data, although sparse, provide additional constraints and help reduce along-path uncertainty (*29*, *30*) because our midpoint assignment assumes uniform sensitivity along the path. This area closely corresponds to the region exhibiting the largest amplitudes of *ScS* pre- and postcursors (diamond, Fig. 2B) (*21*), as well as previously published locations based on *S*_diff_ studies (crosses, Fig. 2B) (*6*, *30*, *31*). The *P*_diff_ observations are crucial for this spatial localization of potential mega-ULVZ locations; the geographic pattern of *S*_diff_ correlations cannot on its own localize the structure because of inherent trade-offs (see fig. S3). The spatial coherence observed strongly indicates a common structural origin at the CMB, effectively excluding source- or receiver-side effects.

**Fig. 3.**
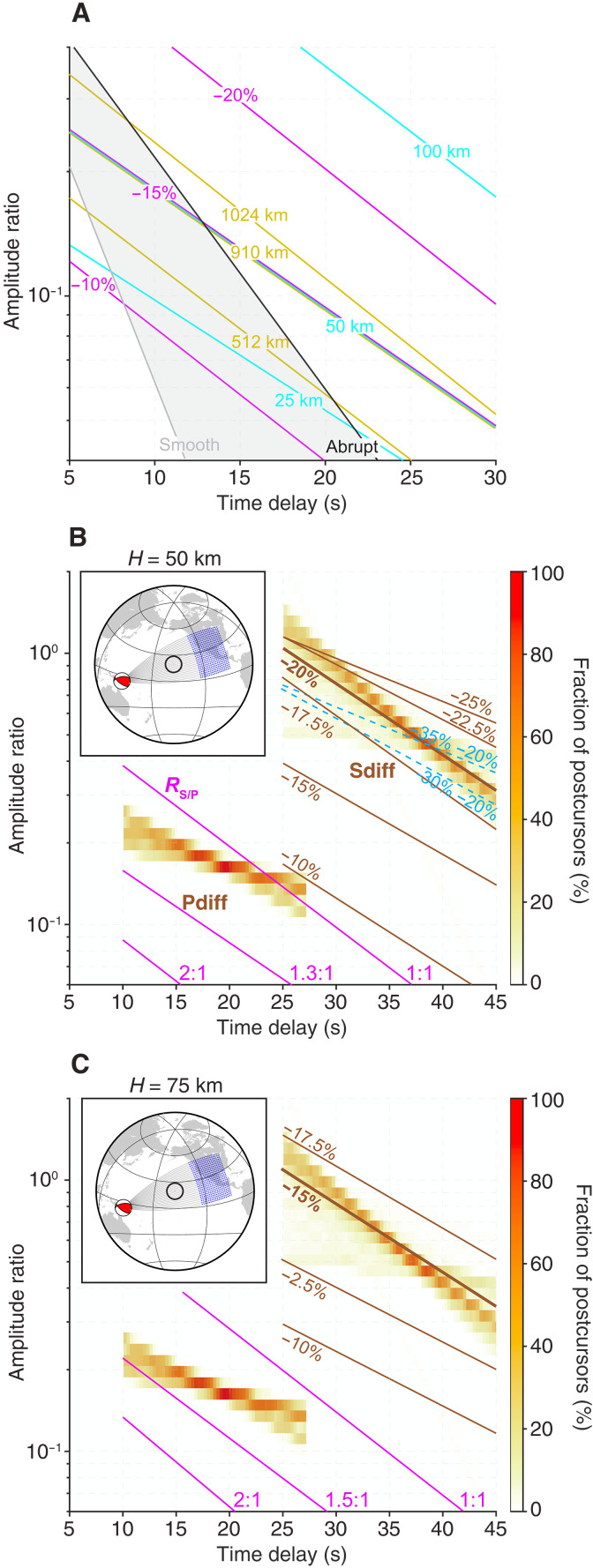
Comparison of observed and predicted postcursor measurements. (**A**) Predicted *P*_diff_ postcursor delay times and log-amplitudes for different cylindrical mega-ULVZ model parameters. Colored lines represent variations in $\delta V_{S}$ (magenta), height (cyan), and diameter (yellow), relative to the reference model from ref. (*12*) ($\delta V_{S}$ = −20%, *W* = 910 km, *H* = 50 km). Black and gray lines show effects of varying the sharpness of the velocity transition at the ULVZ boundary from abrupt to smooth (see fig. S5). (**B**) Observed log-amplitude decay of *P*_diff_ and *S*_diff_ postcursors near the Hawai’ian hot spot. Color shading indicates the fraction of postcursors detected within 5° of each search location using data within the 20° radius around the hot spot. Brown curves show model predictions for varying $\delta V_{S}$, assuming a fixed ULVZ size of *H* = 50 km and *W* = 910 km. The best-fit model highlighted in bold ($\delta V_{S}$ = −20%) guided a systematic search for permissible $\delta V_{P}$ (magenta). A thinner ULVZ model (*H* = 25 km, dashed blue curves) underpredicts the observed amplitudes. The inset shows the simulation geometry with source and receiver locations. (**C**) Same as (B) but for a taller ULVZ with a height of 75 km. See fig. S10 for complementary tests with three regional 1D models.

The observed anticorrelation between the delay time and the log-amplitude of *P*_diff_ postcursors provides a critical piece of evidence supporting the presence of localized P-velocity anomalies at the CMB. Reference (*12*) used this anticorrelation, which is also seen in *S*_diff_ waveforms, as a mega-ULVZ detector across the Pacific basin. To interpret this anticorrelation jointly for *P*_diff_ and *S*_diff_ postcursors, here, we performed synthetic waveform modeling using 3D spectral element simulations, evaluating postcursor behaviors across a suite of cylindrical mega-ULVZ models with constant properties (e.g., fig. S4-S9). We note that the assumed cylindrical geometry represents an idealized scenario chosen to provide a tractable framework for joint *P*- and *S*-wave analysis; geodynamical studies suggest more complex morphologies (*16*, *32*)– such as sloped-edge or ridge-like structures- that may also host variable properties with depth or laterally. Our simulations indicate that postcursor amplitudes primarily depend on ULVZ height, width, and velocity reductions ($\delta V_{P}$ and $\delta V_{S}$) (Fig. 3A). While the slope of amplitude decay mainly reflects geometric spreading and seismic attenuation, the velocity gradient sharpness at ULVZ boundaries strongly impacts later-arriving postcursor amplitudes. However, these parameters cannot be uniquely constrained by the postcursor amplitudes and delay times because of the strong trade-off among them; Fig. 3A illustrates this trade-off for *P*_diff_ postcursors. Density variations, however, have a negligible influence on postcursor signals (fig. S9).

Using the mega-ULVZ geometry proposed by ref. (*12*) ($\delta V_{S}$ = −20%, *W* = 910 km, *H* = 50 km), our joint analysis constrains the permissible range of $\delta V_{P}$ between −20 and −15%, corresponding to an *R*_S/P_ of 1 to 1.3 (Fig. 3B). Deviations outside this range substantially degrade fits to observed amplitudes and delay times; for instance, reducing or increasing $\delta V_{P}$ by 10% either weakens amplitudes or significantly delays postcursor arrivals, respectively. At reductions weaker than 10%, synthetic *P*_diff_ postcursors become nearly undetectable.

Alternatively, a taller ULVZ model (*H* = 75 km) with smaller wave speed reductions ($\delta V_{P}$ = −15 to −10%, $\delta V_{S}$ = −15%) could also explain the observed amplitudes and delay times, yielding a slightly broader but compatible *R*_S/P_ range of 1 to 1.5 (Fig. 3C). In contrast, thinner ULVZ models (*H* = 25 km) fail to reproduce observed data (blue dashed, Fig. 3B). Our modeling reveals critical insight on ULVZ heights: As the height decreases, the absolute velocity reductions required to match observed postcursors increase differently for *P* and *S* waves. Beyond a certain threshold, further velocity reductions saturate postcursor amplitudes of *S* waves, instead enhancing late-arriving phases that are not observed. This implies that accounting for differences in depth sensitivity between *P*_diff_ and *S*_diff_ postcursors—even when measured at comparable wavelengths (or periods, in our case, 6.7 and 17 s, respectively)—is crucial for accurate inferences of *R*_S/P_.

Ultimately, our simulations highlight the need for complementary seismic datasets, such as *ScS* waves (*21*), which have favored thinner ULVZ estimates, to constrain ULVZ size effectively and mitigate inherent modeling trade-offs. Constraints on *R*_S/P_ in Hawai’i have so far been limited because of a preponderance of single-phase studies. The only published constraints come from 2D waveform modeling of high-frequency *P*_diff_ and lower-frequency *S*_diff_ postcursors (*25*) and argue for *R*_S/P_ in the 1.3-to-2 range for a 25-km-tall mega-ULVZ beneath Hawai’i. This upper bound exceeds our upper bound on an *R*_S/P_ of 1.3 for a 50-km-tall structure. According to our 3D modeling results, reproducing an *R*_S/P_ of 2 would require substantially taller ULVZs (exceeding 75 km). This discrepancy likely arises from limitations inherent to 2D modeling, which cannot fully capture multipathing and out-of-plane scattering effects crucial in postcursor modeling. Alternatively, it may imply that *R*_S/P_ increases within the mega-ULVZ toward the CMB. Thus, fully 3D wavefield simulations spanning the frequency range of observational constraints are essential for furthering our insights into the internal ULVZ structure from *P*_diff_ and *S*_diff_ postcursor observations.

*P*_diff_’s broader sensitivity to the lower-mantle structure raises the possibility that features beyond a localized ULVZ could contribute to the observed postcursors. For example, a few anomalously large-amplitude *P*_diff_ signals southeast of Hawai’i (Fig. 2A)—absent in the *S*_diff_ dataset—might suggest such an influence. We therefore evaluated models involving LLVP tops and large-scale velocity gradients (fig. S10). Underside reflections from LLVP tops (*33*, *34*) can in principle produce strong *P*_diff_ postcursors, but they predict both delay time and amplitude increasing with distance, opposite to our observations (Fig. 3). Models with large negative velocity gradients in the lowermost mantle (*35*) generate signals that are far too weak. Similarly, *S*_diff_ has shown only weak and diffuse scattered signals from LLVP boundaries, appearing pervasively across the Pacific basin without a clear relationship between delay-time variations and postcursor amplitude (*12*).

## DISCUSSION

**Fig. 4.**
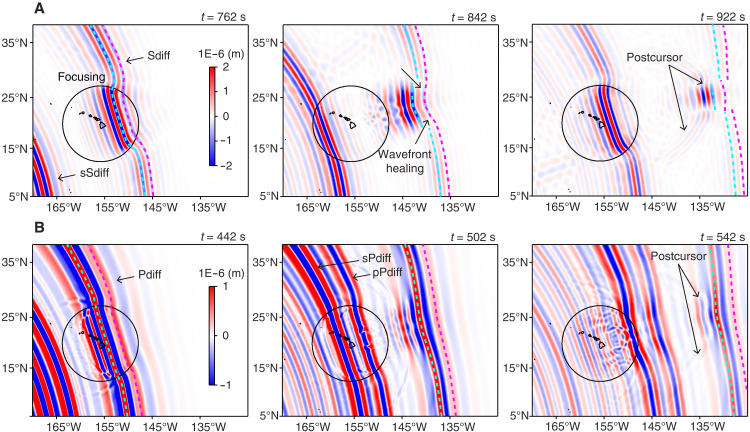
Time evolution of 3D wavefields illustrating the propagation of the diffracted wavefront. Snapshots of map views of (**A**) *S*_diff_ wavefront propagation at time intervals, zooming in on the interaction with an ULVZ. The strongest postcursor amplitudes arise from the main diffracted wavefront (purple), highlighting the roles of wavefront healing (cyan) and focusing together in shaping amplitude variations and travel time delays, particularly when waves fully transmit through the ULVZ. (**B**) Same as (A) but for *P*_diff_ wavefront propagation. Because of the faster background *P*-wave velocity and the smaller magnitude of $\delta V_{P}$ relative to the *S* wave, wavefront healing occurs rapidly. As a result, the main diffracted wavefront contributes marginally to postcursor amplitudes, leading to postcursor amplitudes that are much smaller than for *S*_diff_.

As discussed by ref. (*6*), ray theory suggests that waves fully transmitting through the ULVZ would be the most slowed down, although their delay is reduced in comparison to waves refracting around the ULVZ because the latter accumulate additional off-great-circle propagation time. While multipathing is generally invoked to explain postcursor travel time delays as a function of azimuth, a 3D wavefield propagation analysis provides additional insights. For *S* waves, our time-evolution analysis of the 3D wavefield reveals that the earliest-arriving (smallest delay time) postcursor originates from the main diffracted wavefront, which is delayed, forming postcursors (Fig. 4A), and amplified as a result of focusing. Simultaneously, what has been typically interpreted as the main *S*_diff_ or *P*_diff_ phase is actually produced by wavefront healing of waves diffracting around the anomaly, a phenomenon that is unaccounted for by ray theory. This wavefront healing does not fully erase the wavefront disruption caused by passage through the ULVZ, resulting in a first-arriving *S*_diff_ (*P*_diff_) phase that is smaller in amplitude than it would have been without the interaction with the ULVZ. This further enhances the postcursor–to–main phase ratio, contributing to the very large amplitude of *S*_diff_ postcursors beneath Hawai’i, which can exceed those of the main *S*_diff_ phase (see Fig. 1F). Waves scattered by the ULVZ without traveling through it have small amplitudes, consistent with forward scattering effects as they propagate away from the ULVZ center. For *P* waves, the wavefield simulations (Fig. 4B) reveal that wavefront healing is more effective in erasing the wavefront disruption caused by passage through the ULVZ, resulting in a smaller postcursor–to–main phase ratio. This amplitude ratio is further reduced by the less effective focusing and smaller separation between the postcursor and the main phase, owing to the smaller magnitude of $\delta V_{P}$ and the faster background *P*-wave speed. This explains the relatively small amplitudes of *P*_diff_ postcursor observation.

**Fig. 5.**
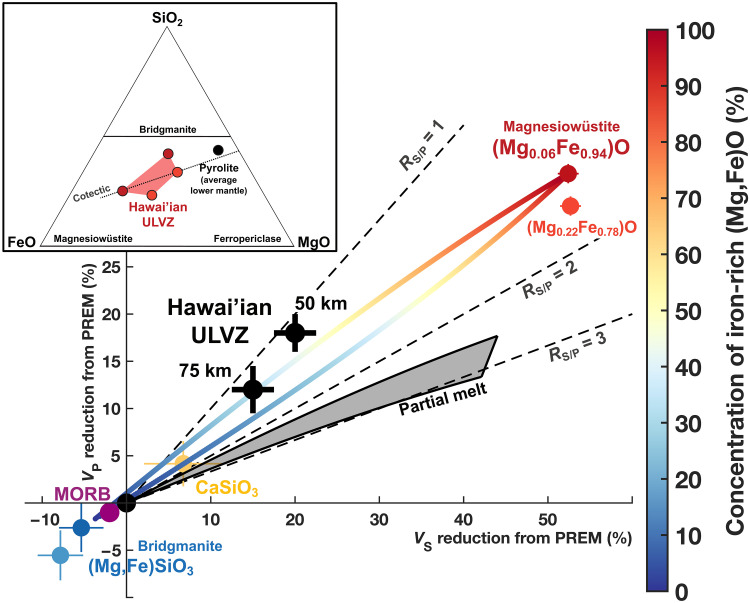
Seismic velocity variations from the average lowermost mantle (PREM) for relevant minerals and rocks. Iron enrichment in pyrolite-like mantle rocks produces assemblages containing iron-rich (Mg,Fe)O coexisting with (Mg,Fe)SiO_3_ and CaSiO_3_. These solid assemblages exhibit bulk seismic velocities (colored lines) compatible with the seismic models (upper line, Voigt bound; lower line, Reuss bound). Partial melting of ambient mantle or subducted oceanic crust [MORB (mid-ocean ridge basalt)] is incompatible with the seismic models. Mineral and rock seismic properties are reported in table S1. Examples of solid iron-rich rocks that represent approximate bounds on viable ULVZ compositions are reported in table S2 and shown in the inset. This compositional range falls close to the cotectic line that represents the evolution of a crystallizing basal magma ocean (*49*, *50*).

The joint constraints on both *P*- and *S*-wave speeds of the Hawai’ian mega-ULVZ allow us to evaluate major hypotheses for ULVZ composition and origin. In particular, we compare our seismically constrained *R*_S/P_ values with plausible mineralogical models that consider both iron enrichment and partial melting in the lowermost mantle (table S1). We find that solid iron-enriched lower mantle rocks, containing magnesiowüstite [(Mg,Fe)O] phases (*36*) close to the FeO end-member [e.g., (Mg_0.06_Fe_0.94_)O] coexisting with bridgmanite [(Mg,Fe)SiO_3_] and CaSiO_3_, provide the best match to the seismic models (Fig. 5). A range of (Mg,Fe)O concentrations can reproduce the seismic models (table S2) depending on the distribution of stress among the mineral phases, bounded by the Voigt and Reuss mixing cases (*37*, *38*). Lower levels of iron enrichment [e.g., (Mg_0.22_Fe_0.78_)O] yield slightly poorer fits to the seismic model but remain within acceptable limits given mutual uncertainties (fig. S11). Small degrees of partial melting (e.g., 1%) in such iron-enriched rock are acceptable but do not provide a better fit to the seismic models (fig. S12). On the other hand, partial melting of the pyrolitic lowermost mantle or the subducted oceanic crust does not appear consistent with the inferred seismic wave speed reductions (Fig. 5). Infiltration of liquid from the outer core would similarly produce *R*_S/P_ values that are too high to match the seismic models.

Localized iron enrichment in the form of mega-ULVZs may further affect upwelling dynamics because of the metallic behavior of FeO under mantle base conditions. Experimental (*39*) and theoretical (*40*) findings on FeO point to high electrical conductivities that would imply elevated thermal conductivity in FeO-rich rock. Iron-rich mega-ULVZs could therefore actively promote localized long-term plume generation (*41*) and influence long-wavelength convection in the mantle, instead of serving only as passive markers of hot upwelling regions. Conversely, some recent seismic studies have reported the presence of ULVZs in regions of active subduction (*42*, *43*) or far from active downwelling or upwelling (*44*). These studies generally report *R*_S/P_ closer to 3 or 4, notably higher than the ratios we infer for the Hawai’ian mega-ULVZ and more consistent with the presence of partial melting (Fig. 5). Subduction-associated ULVZs may represent regions of active partial melting of oceanic crust or perhaps reaction of hydrous material with core fluid. Both processes could induce the local enrichment of FeO (*45*) that may, in the long term, feed a global basal layer that serves as source material for FeO-rich mega-ULVZs.

We conclude that a solid-state Hawai’ian mega-ULVZ containing iron-rich (Mg,Fe)O is the preferred explanation for our seismic observations. Previous geodynamic simulations have shown that a thin basal layer of solid dense rock can be swept up by mantle convection to form stable structures with ULVZ-like morphologies (*16*, *46*, *47*), while dense partial melt may more likely drain out from the ULVZ (*15*). Recent work using normal mode frequency observations (*48*) reported the existence of such a global, kilometer-scale layer with seismic velocities and densities compatible with iron-rich rock or partial melt. We further note that the iron-rich rocks modeled here have compositions lying around the cotectic line predicted for partial melting or fractional crystallization of the pyrolitic lower mantle (inset, Fig. 5) (*49*, *50*). The source material for the Hawai’ian mega-ULVZ may thus be a relic of fractional crystallization of a basal magma ocean (*51*) or recrystallization products of iron-rich melts formed by prior episodes of partial melting in the lowermost mantle. Another possibility is that the reaction of hydrous slab debris with outermost core fluid would induce FeO enrichment at the CMB (*45*). In some Hawai’ian lavas, ancient negative μ^182^W isotopic signatures are observed to be strongly correlated with primitive mantle material contributions (i.e., elevated ^3^He/^4^He ratios) (*8*); these isotopic anomalies can be explained if Hawai’ian lavas sample traces of ULVZ material that has experienced isotopic exchange with the core (*52*), perhaps in the basal magma ocean stage or in subsequent slab-core interactions.

## MATERIALS AND METHODS

### Seismic waveform sequencing

We used vertical-component *P*_diff_ seismic waveforms of *M*_w_ > 6.5 earthquakes between 100° and 110° recorded by broadband seismic stations operating during 1990 to 2021. All earthquake waveform data are available through the IRIS Data Management Center (https://ds.iris.edu/ds/nodes/dmc/). The *S*_diff_ waveforms, taken from previous work (*12*), span 1990 to 2018 and therefore include 12 fewer events than the full set of 183 earthquakes initially considered. The instrument response was removed from the raw data, and the resulting displacement seismograms were bandpass filtered between 5 and 100 s for *P*_diff_ and 15 and 100 s for *S*_diff_ waveforms using a fourth-order Butterworth filter. To minimize the effects of source radiation pattern resulting from variations in focal mechanisms, the waveforms were deconvolved using synthetic waveforms computed for each event’s centroid moment tensor (*53*) and PREM (*26*) using Instaseis (*27*).

After preprocessing the waveform data, we systematically identified *P*_diff_ postcursor signals by arranging our preprocessed *P*_diff_ waveform data using a graph-based, 1D manifold learning approach called the Sequencer (*28*). This method allowed us to contextualize full waveforms (i.e., amplitude and timing of seismic arrivals) by optimally ordering waveforms in a way that maximizes the total similarity between adjacent reordered waveforms. Sequencing was performed across various time windows (scales), and for each scale, we quantified the presence of a dominant trend by measuring the elongation of the minimum spanning tree derived from the corresponding distance matrix. The Sequencer aggregates information from all relevant scales and similarity metrics to define a final ordering of the data that maximizes this elongation, thereby identifying the strongest single trend in the dataset. Unlike the popular dimensionality reduction algorithm t-SNE (t-distributed stochastic neighbor embedding) (*54*), our approach is fully deterministic and can isolate scales or subsets of data that contribute to an underlying trend. For our *P*_diff_ data, we used the Wasserstein (Earth mover) distance as a similarity metric computed across the full waveform. To highlight the *P*_diff_ postcursors and suppress apparent waveform differences resulting from travel-time variations, we aligned the deconvolved waveforms by cross-correlation and removed the mean trace from each waveform before sequencing. We focused on the records following the main *P*_diff_ positive pulse and applied histogram equalization to the data before inputting them into the Sequencer (fig. S1D). The pixel-based histogram equalization applied here is analogous to applying automatic gain control, often used in reflection seismology, to balance amplitudes in seismic records. Histogram equalization redistributes the range of amplitudes in the data, effectively normalizing them in a nonlinear way that enhances the visibility of weaker signals arriving later after the main seismic phase.

After sequencing, we applied a running median filter to compute residual waveforms and discarded noisy waveforms with root-mean-square amplitudes greater than 1 − σ. Restricting the dataset to events deeper than 200 km, with epicentral distances between 100° and 110°, and after applying the above processing steps, we obtained 81 events for *P*_diff_ and 59 for *S*_diff_. The two datasets overlap almost entirely, with only one *S*_diff_ event not represented in the *P*_diff_ set. These final event sets were used for the postcursor analysis described in the main text. The complete lists of these events, along with the seismic networks used in this study and their associated citations, are provided in the supplementary files (data S1and S2).

The geographic distribution of observed *P*_diff_ postcursors was illustrated (Fig. 2A) by plotting each waveform as a circle at the midpoint of its diffracted path along the CMB, colored according to its position in the sequence. For comparison, the previously documented geographic distribution of *S*_diff_ postcursors from the same region (*12*) is presented in fig. S2. To quantify the characteristics of *P*_diff_ postcursors, we analyze the delay time and maximum amplitude of post-*P*_diff_ arrivals relative to the main *P*_diff_ phase using the sequenced ordering of the waveforms (Fig. 1B).

Wavefronts propagating through and around the ULVZs experience folding at caustics, which can introduce a π/2 phase shift and complicate the interpretation of the postcursor waveforms. To account for this phase distortion, we analyze the amplitude of the analytic signal by applying a Hilbert transform to seismic waveforms to calculate the seismic envelopes. The analysis focuses on the first 40-s window following the *P*_diff_ phase to avoid any potential contamination from depth phases associated with *P* waves. Then, we compute the slope and amplitude of the best-fit line to the average delay times and logarithmic amplitudes of the postcursors. The slope and amplitude calculations are made on waveforms corresponding to diffraction paths within a 5° radius of locations distributed systematically across a region within 20° of the Hawai’ian hot spot for robustness (Fig. 3, B and C). This approach is based on the expectation that a localized anomaly at the CMB would result in an anticorrelation between the delay time and the log-amplitude, manifesting as a linear trend with a negative slope (*12*).

### 3D wavefield simulations

To relate the observed relationship between the delay time and the logarithmic amplitude of the postcursors to the properties of the mega-ULVZ beneath Hawai’i, we generated synthetic waveforms using 3D spectral element methods. Two widely used codes, SPECFEM3D (*55*) and AXISEM-3D (*56*), were used in this study. Our waveform simulations resolved periods down to ∼6.3 s for SPECFEM-3D and ∼5 s for AXISEM-3D, compatible with the periods used in the *P*_diff_ data analysis. The modeling domain for SPECFEM-3D consisted of two chunks of the cubed sphere (fig. S4). We ensured that the domain was sufficiently large to encompass the source-receiver geometry used in the modeling. Laterally, each chunk was subdivided into 480 elements in the crust and upper mantle, gradually coarsening to 120 elements at the CMB, with an average spacing of ∼45.6 km. Absorbing boundary conditions were implemented at the edges of the model space to minimize unwanted reflections. In AXISEM-3D, 3D simulations were achieved by rotating a 2D meridian domain, discretized using a finite-element mesh, into a spherical domain with a D-shaped mesh. AXISEM-3D incorporates a Fourier basis for azimuthal expansion of the wavefield, allowing specification of a maximum Fourier expansion order, ν. To ensure accuracy, we verified that all output ν values remain below the maximum constant defined by the wavefield learning tool (*56*), with typical ν values exceeding ∼700.

We introduced a cylindrical mega-ULVZ at the base of the mantle (green, fig. S4) using the PREM as the background. Parameter ranges considered in our modeling included $\delta V_{S}$ (−10 to −35%), *R*_S/P_ (1 to 3), width (*W*, 512 to 1024 km), and height (*H*, 25 to 100 km) with a constant density increase of 10%. In addition, we tested models with smooth velocity transitions across boundaries using cosine tapers (fig. S5). While more complex ULVZ morphologies have been proposed (*57*), the simplified cylindrical models we used reduced the parameter space, enabling a systematic analysis of individual parameter influences on postcursor measurements and their trade-offs. Moment tensor solutions for each event used in the modeling were obtained from the Global Centroid Moment Tensor catalog (*53*). Time-evolution snapshots of the wavefield were recorded to examine wave-ULVZ interactions responsible for generating postcursors (Fig. 4). While the primary modeling analysis in this study focused on results from SPECFEM-3D, outputs from AXISEM-3D served as benchmarks. We confirmed that both approaches produced nearly identical results (e.g., figs. S6 to S8), validating the consistency of our modeling. Synthetic waveforms corresponding to each ULVZ model were processed using the same procedures described in the preceding section. In addition to the ULVZ simulations, we also tested three regional 1D background models (*33*–*35*) to evaluate the influence of velocity structure on predicted postcursor properties (fig. S10).

### Mineralogical modeling

We evaluated the possible mineralogy of the observed ULVZ using elasticities and densities for multiple compositions of iron-rich (Mg,Fe)O and (Mg,Fe)SiO_3_, as well as calcium silicate perovskite CaSiO_3_. Properties of individual phases and mineral aggregates were calculated for approximate lowermost mantle conditions (136 GPa and 3800 K) using previously published approaches (*38*, *58*) or otherwise taken directly from published studies, as listed in table S1. Partitioning of iron between (Mg,Fe)O and (Mg,Fe)SiO_3_ was computed using a fixed exchange coefficient *K*_D_ = 0.03 on the basis of previous experiments on iron-rich bulk compositions (*59*, *60*). As previously discussed, aggregate properties were calculated for both Voigt and Reuss mixing bounds, which can yield substantially different estimates of bulk properties at a given composition given the large difference in elastic moduli between the seismically slow (Mg,Fe)O and the other phases (*38*). Four example rock compositions, representing approximate bounds on viable ULVZ compositions compatible with seismic models within mutual uncertainties, are reported in table S2 and the inset of Fig. 5.

We also evaluated the effect of partial melting on seismic velocities by following a procedure (*61*) that investigates the effect of melt geometry and composition on velocity reductions. Melt geometries, defined by the dihedral angle θ of the melt pocket, are poorly constrained under CMB conditions. Experiments on an iron-nickel-sulfur alloy in an enstatitic assemblage showed a decrease in dihedral angle from θ > 60° below 40 GPa (corresponding to isolated melt pockets) to θ ∼ 20° at 64 GPa [corresponding to interconnected melt networks (*62*)]. In our analysis, we assumed a fixed dihedral angle θ ∼ 20° for the melt and calculated seismic velocity reductions, given three hypothetical liquid compositions—outermost core liquid from PREM, liquid FeO (representing the extreme case of iron enrichment in the melt) (*63*), and a liquid with elasticity identical to the coexisting solid (approximating equal levels of iron concentration in the solid and melt). In fig. S12, we illustrate the effects of melt geometry and composition on seismic velocity reductions.

## Supplementary Materials

### The PDF file includes:

Figs. S1 to S12 Tables S1 and S2 Legends for data S1 and S2 References
