# Supplementary material for: Seismic and mineralogical evidence for an iron-rich mega–ultralow-velocity zone beneath Hawai’i
Source: Sci Adv. 2026 Jan 28;12(5):eadz1962. doi: 10.1126/sciadv.adz1962 (PMC12851025; doi:10.1126/sciadv.adz1962)
Supplement: Supplementary file 1 — Figs. S1 to S12 Tables S1 and S2 Legends for data S1 and S2 References [file sciadv.adz1962_sm.pdf]

Science Advances
AAAS

Supplementary Materials for

Seismic and mineralogical evidence for an iron-rich mega–ultralow-velocity zone beneath Hawai'i

Doyeon Kim *et al.*

Corresponding author: Doyeon Kim, doyeon.kim@imperial.ac.uk

Sci. Adv. **12**, eadz1962 (2026)
DOI: 10.1126/sciadv.adz1962

The PDF file includes:

Figs. S1 to S12
Tables S1 and S2
Legends for data S1 and S2
References

Other Supplementary Material for this manuscript includes the following:

Data S1 and S2

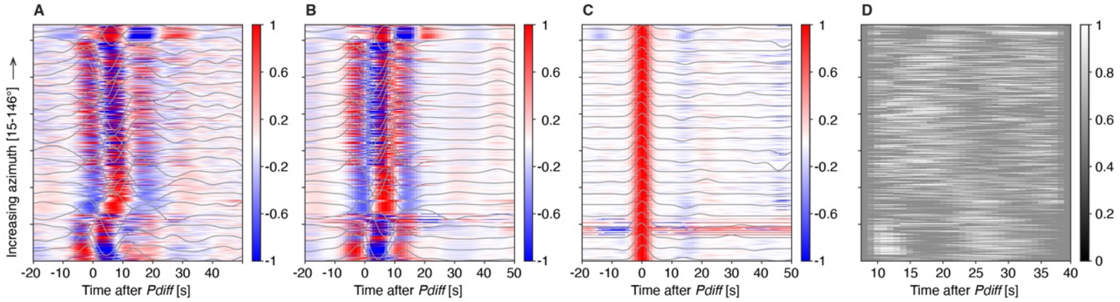

fig. S1. Processing of Pdiff waveforms. (**A**) Raw vertical displacement seismograms and (**B**) synthetic waveforms computed for PREM. The red-blue color scale shows normalized amplitude relative to the main Pdiff arrival. (**C**) Source deconvolved seismograms obtained by taking (A) and deconvolving with (B). (**D**) Waveforms in (C) applied with the histogram equalization, used as input for the Sequencer.

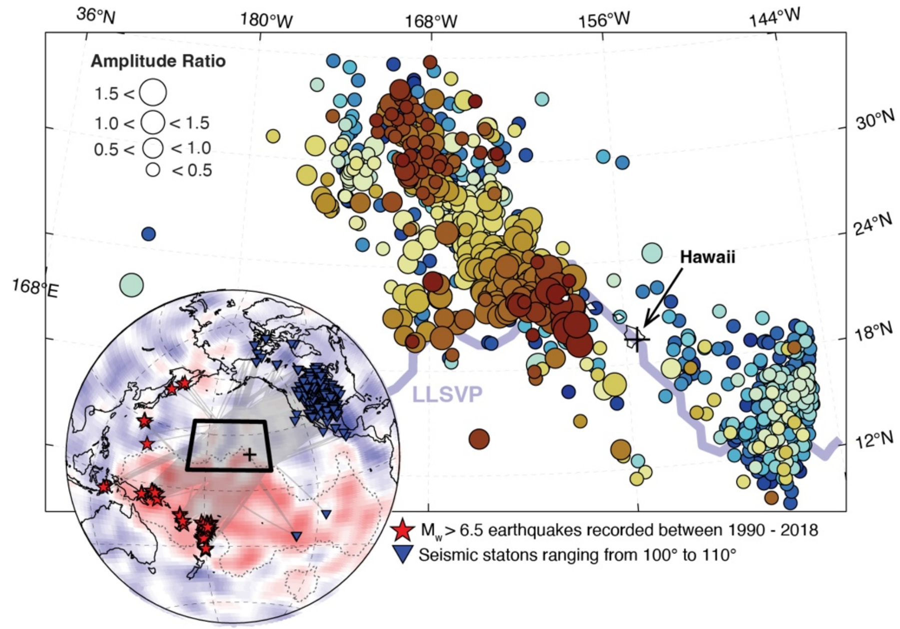

fig. S2. Spatial distribution of the Sdiff postcursors in Hawai'i (after (*12*)**)**. Inset map shows the locations of earthquakes (red) and seismic stations (blue) used in the study, along with the geographic extent of the Pacific LLVP, marked by the transition from red to blue regions.

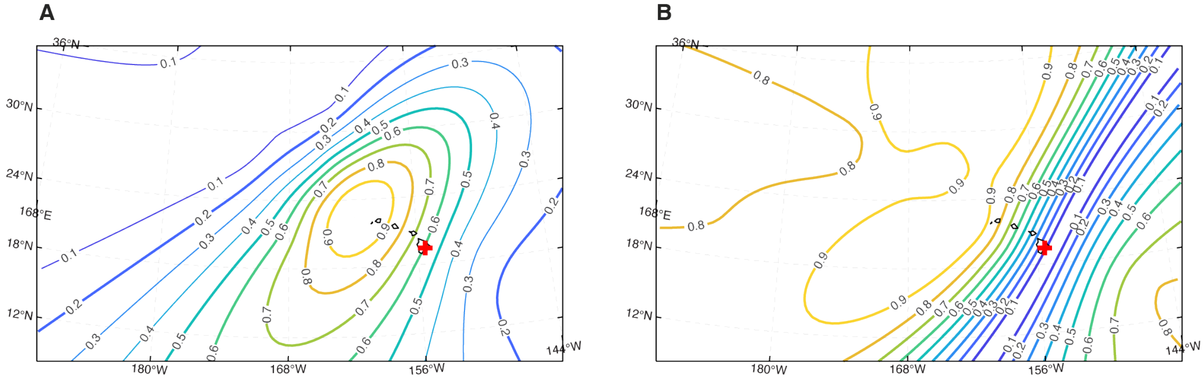

fig. S3. Correlation coefficients between sequenced index and midpoint distance from putative mega-ULVZ locations. Contour plot of the correlation between sequenced index and path-midpoint distance associated with each potential location of the mega-ULVZ for combined Pdiff (**A**) and Sdiff (**B**) datasets. At each location, distance is computed to all the midpoints for each diffracted path in the dataset and then the correlation coefficient is computed between that distance and the sequenced index. The product of the Pdiff and Sdiff correlation coefficients is shown in Fig. 2A). For these computations, only data exhibiting clear postcursors (the latter half of the indices) were included, while waveforms without postcursor signals were excluded.

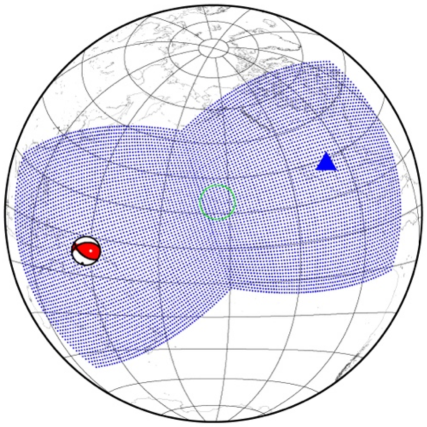

fig. S4. SPECFEM-3D modeling domain. SPECFEM-3D Globe spectral element grids, illustrating the model domain with two chunks covering the major source and receiver regions relevant to the dataset. The reference source parameters are based on the CMT solution (*53*) of M_W6.9 earthquake in the Eastern New Guinea region on April 17, 2012, with a hypocentral depth of 208.6 km. Multiple point-profile models of the ULVZ are tested using the 1-D transversely isotropic PREM as the background. The primary synthetic waveforms and analyses are derived from SPECFEM-3D outputs, verified with additional simulations performed using AXISEM-3D (e.g., Fig.S6-S8.)

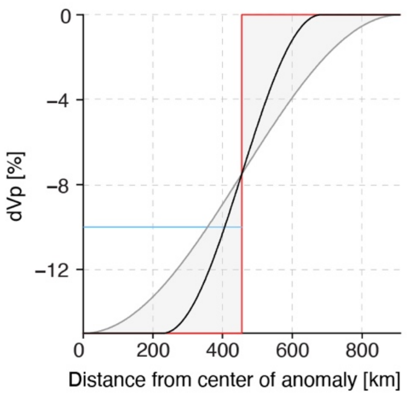

fig. S5. ULVZ models with different edge sharpness in velocity. The effect of velocity gradient sharpness at the edges of ULVZ models is tested by applying a cosine taper to create smooth edges. The shaded region illustrates the transition from abrupt to smooth model boundaries, with red and blue lines representing synthetic measurements corresponding to those plotted in Fig. 3A.

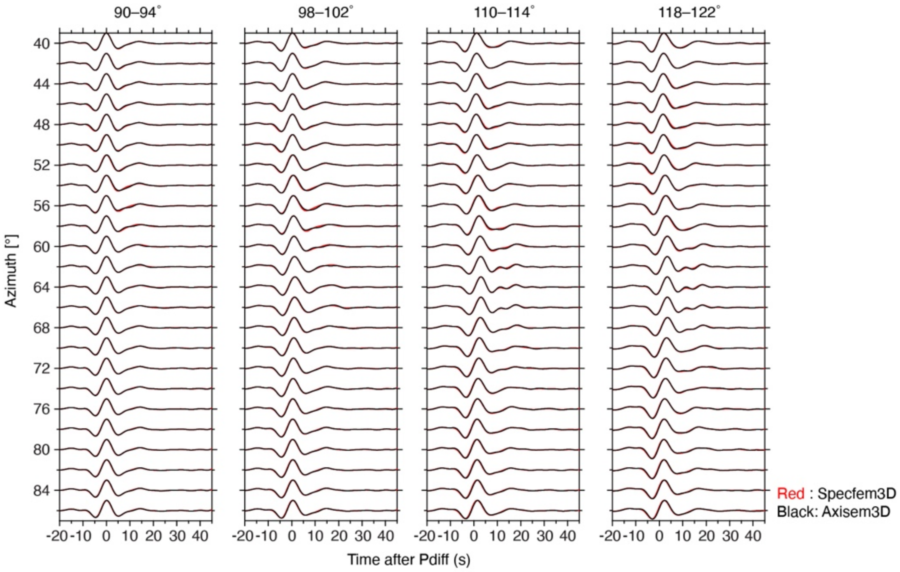

fig S6. Synthetic Pdiff waveforms. Comparison of synthetic waveforms computed based on two different spectral element methods employed in this study. The model includes a cylindrical ULVZ structure with *H* = 50 km, *W* = 910 km, *dVp* = -10%, and *dVs* = -20%, embedded within the PREM background. The epicentral distance ranges of the receivers that record the synthetic seismograms are shown above each panel.

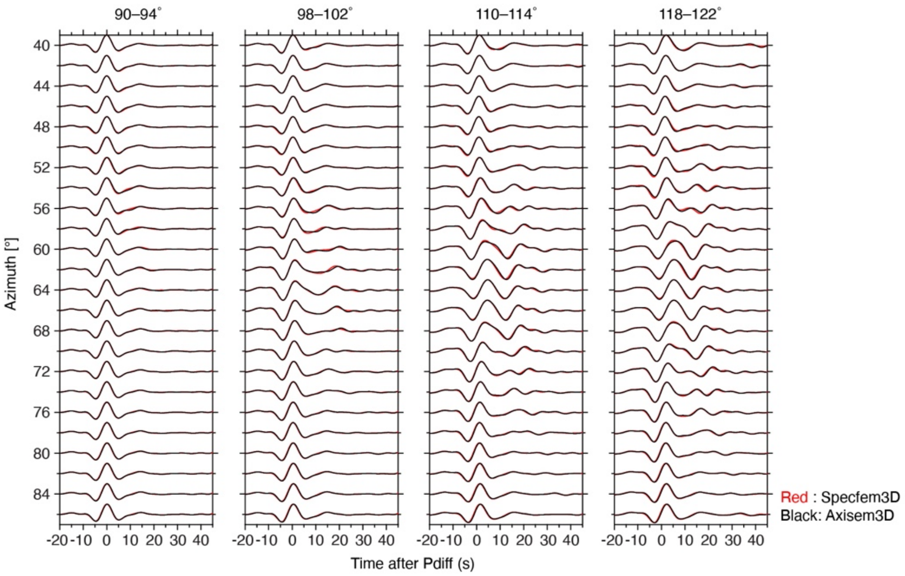

fig. S7. Synthetic Pdiff waveforms. Same as fig. S6 but using a *dVp* = -20%.

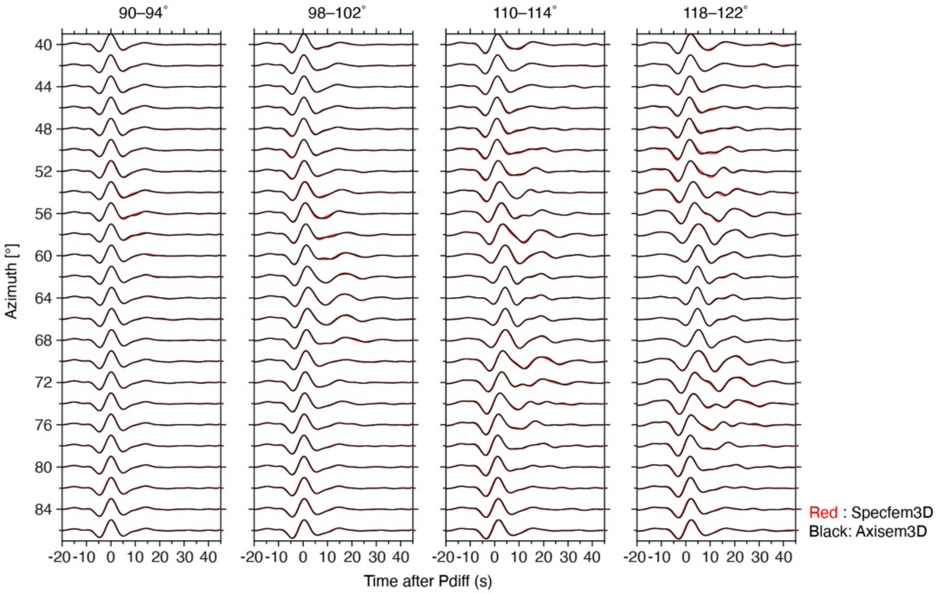

fig. S8. Synthetic Pdiff waveforms. Same as fig. S6 but using a H = 100 km.

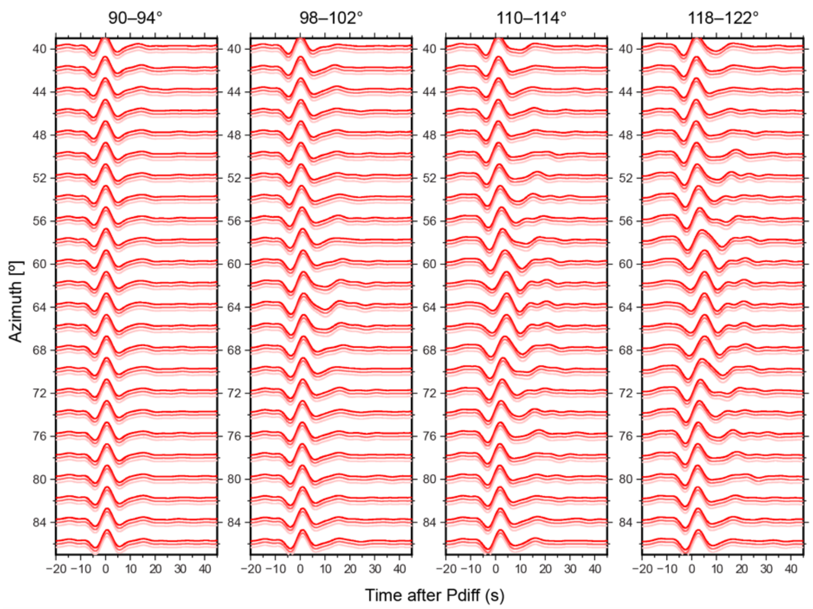

fig. S9. Synthetic Pdiff waveforms. Comparison of synthetic waveforms for a cylindrical ULVZ structure (as fig. S6), but with varying density (pale red: Δρ = +0%, medium red: Δρ = +10%, bright red: Δρ = +15). Waveforms are computed with SPECFEM-3D. Waveforms are computed using SPECFEM-3D and are slightly offset by azimuth for clarity. Differences between the waveforms are negligible.

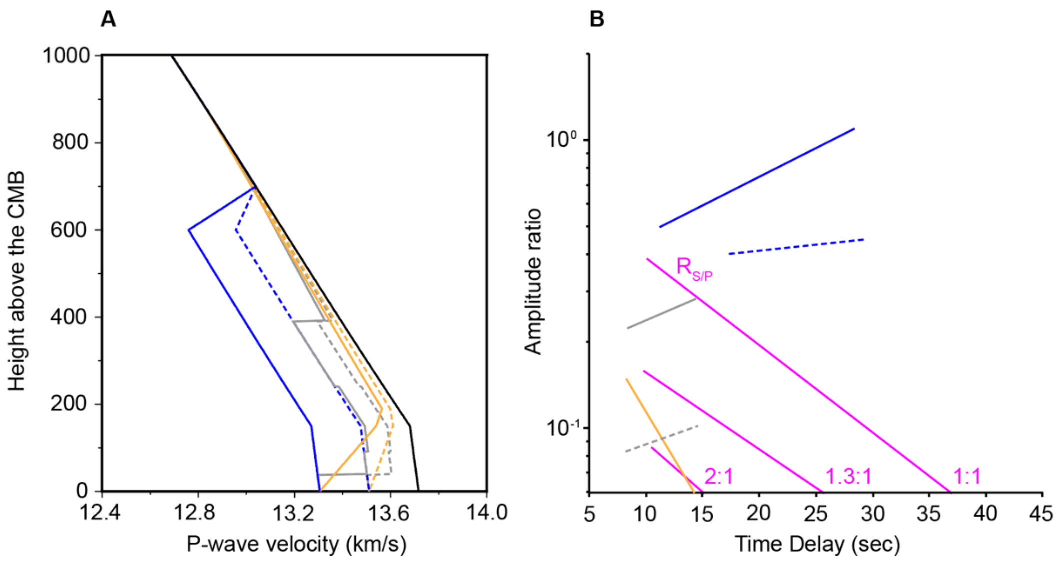

fig. S10. Predicted Pdiff postcursor delay times and log-amplitude ratios for three regional 1-D models. (A) P-wave velocity profiles used for waveform simulations. (B) Corresponding synthetic delay times and log-amplitude measurements (gray: *33*; blue: *34*; orange: *35*). The velocity profiles are derived from original S-wave velocity models, modified using $R_{S/P}$ = 1:1 (solid lines) and 2:1 (dashed lines). Magenta lines indicate predictions from the cylindrical ULVZ model with H = 50 km with varying $R_{S/P}$ (Fig. 3B). Synthetic Pdiff postcursors from the red dashed model are too weak to be plotted.

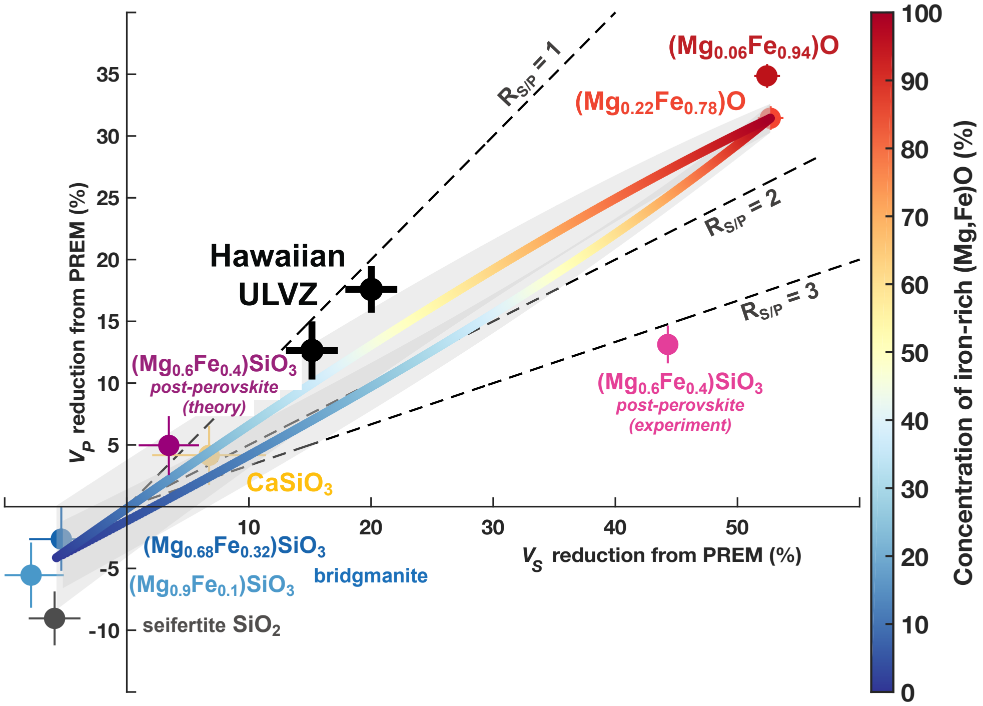

fig. S11. Effect of lower iron content on bulk rock sound velocities. Shaded regions indicate uncertainties on the aggregate sound velocities. Sound velocity values and references for individual phases are listed in table S1.

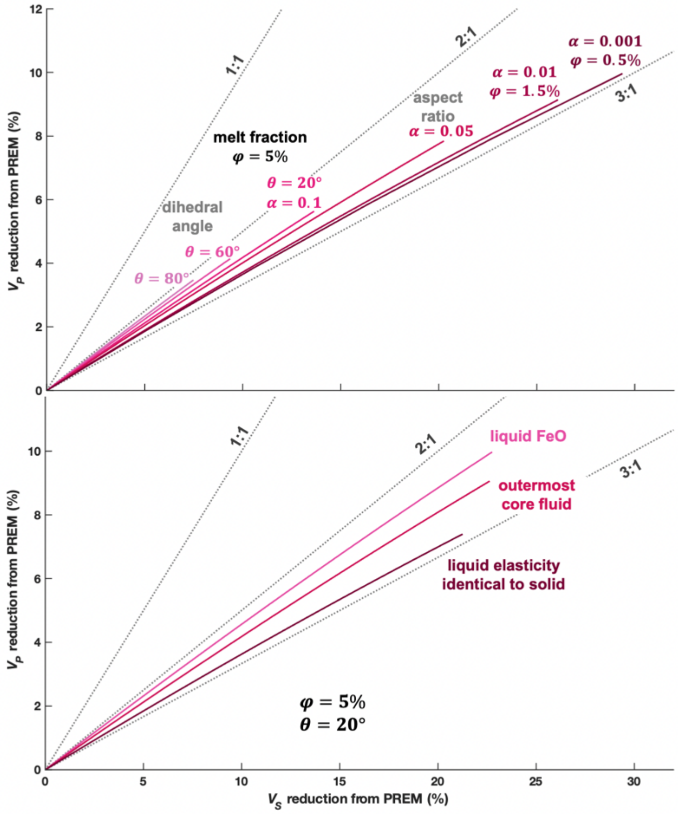

fig. S12. Sound velocity reductions in partially molten rock. Forward models showcase examples of how melt geometry, fraction, and composition affect the relative reductions in P and S wave speeds.

table. S1. Mineral and rock properties used for mineralogical models. Values are previously calculated for core-mantle boundary conditions (*38,59*) or taken from published studies (*64–67*).

**Phase**	V_P **(km/s)**	V_S **(km/s)**	ρ **(g/cm³)**
$(Mg_{0.22}Fe_{0.78})O$ magnesiowüstite "Mw78"	9.40 ± 0.14	3.44 ± 0.09	7.89 ± 0.02
$(Mg_{0.06}Fe_{0.94})O$ magnesiowüstite "Mw94"	8.93 ± 0.14	3.46 ± 0.09	8.54 ± 0.02
$(Mg_{0.90}Fe_{0.10})SiO_3$ bridgmanite "Br10"	14.47 ± 0.36	7.84 ± 0.20	5.42 ± 0.05
$(Mg_{0.68}Fe_{0.32})SiO_3$ bridgmanite "Br32"	14.07 ± 0.35	7.66 ± 0.19	5.73 ± 0.06
$CaSiO_3$	13.14 ± 0.33	6.78 ± 0.34	5.49 ± 0.05
$(Mg_{0.60}Fe_{0.40})SiO_3$ post-perovskite (experiment)	11.91 ± 0.21	4.05 ± 0.03	5.90 ± 0.06
$(Mg_{0.60}Fe_{0.40})SiO_3$ post-perovskite (theory)	13.03 ± 0.33	7.02 ± 0.18	5.90 ± 0.06
SiO_2 seifertite	14.95 ± 0.30	7.70 ± 0.15	5.45 ± 0.10
Mid-ocean ridge basalt (MORB)	13.85 ± 0.15	7.42 ± 0.07	5.62 ± 0.11

table. S2. Properties of iron-rich mineral assemblages that reproduce the seismic models. The four compositions are identical to those plotted in the inset of Fig. 5 and represent approximate bounds on compositions that exhibit sound velocities compatible with seismic models, within uncertainties. $R_{S/P}$ values for each composition are listed as ranges that account for uncertainties in the seismic velocities of the mineral assemblage, as showcased by the shaded lines in Fig. S12.

**Property**	**Mw94+Br32** (Reuss, h = 75 km)	**Mw94+Br32** (Voigt, h = 50 km)	**Mw78+Br10** (Voigt, h = 75 km)	**Mw78+Br10** (Voigt, h = 50 km)
X_{Br} (%)	84	50	67	46
X_{Mw} (%)	16	50	33	54
$\delta \ln \rho$ (%)	10	28	12	21
$R_{S/P}$	1 – 2.4	1.2 – 1.7	1.2 – 2.3	1.3 – 1.9
FeO (mol%)	28.5	55	29.1	44.4
MgO (mol%)	29.5	20	37.4	32.6
SiO_2 (mol%)	42	25	33.5	23

Data S1. (separate file)

Table of earthquake events used in the study.

Data S2. (separate file)

List of seismic networks providing data, with corresponding citations.

REFERENCES

1. E. J. Garnero, A. K. McNamara, S.-H. Shim, Continent-sized anomalous zones with low seismic velocity at the base of Earth's mantle. *Nat. Geosci.* **9**, 481–489 (2016).

2. V. Lekic, S. Cottaar, A. Dziewonski, B. Romanowicz, Cluster analysis of global lower mantle tomography: A new class of structure and implications for chemical heterogeneity. *Earth Planet. Sci. Lett.* **357**, 68–77 (2012).

3. S. Yu, E. J. Garnero, Ultralow velocity zone locations: A global assessment. *Geochem. Geophys. Geosyst.* **19**, 396–414 (2018).

4. M. S. Thorne, E. J. Garnero, G. Jahnke, H. Igel, A. K. McNamara, Mega ultra low velocity zone and mantle flow. *Earth Planet. Sci. Lett.* **364**, 59–67 (2013).

5. E. J. Garnero, J. Revenaugh, Q. Williams, T. Lay, L. H. Kellogg, "Ultralow velocity zone at the core-mantle boundary" in *The Core-Mantle Boundary Region* (American Geophysical Union, 1998), vol. 28, pp. 319–334.

6. S. Cottaar, B. Romanowicz, An unsually large ULVZ at the base of the mantle near Hawaii. *Earth Planet. Sci. Lett.* **355**, 213–222 (2012).

7. S. Mukhopadhyay, Early differentiation and volatile accretion recorded in deep-mantle neon and xenon. *Nature* **486**, 101–104 (2012).

8. A. Mundl, M. Touboul, M. G. Jackson, J. M. Day, M. D. Kurz, V. Lekic, R. T. Helz, R. J. Walker, Tungsten-182 heterogeneity in modern ocean island basalts. *Science* **356**, 66–69 (2017).

9. C. D. Williams, S. Mukhopadhyay, M. L. Rudolph, B. Romanowicz, Primitive helium is sourced from seismically slow regions in the lowermost mantle. *Geochem. Geophys. Geosyst.* **20**, 4130–4145 (2019).

10. K. Yuan, B. Romanowicz, Seismic evidence for partial melting at the root of major hot spot plumes. *Science* **357**, 393–397 (2017).

11. S. Cottaar, C. Martin, Z. Li, R. Parai, The root to the Galapagos mantle plume on the core-mantle boundary. *Seismica* **1**, 10.26443/seismica.v1i1.197 (2022).

12. D. Kim, V. Lekic, B. Menard, D. Baron, M. Taghizadeh-Popp, Sequencing seismograms: A panoptic view of scattering in the core-mantle boundary region. *Science* **368**, 1223–1228 (2020).

13. Z. Li, C. Martin, S. Cottaar, Seismic observation of a new ULVZ beneath the Southern Pacific. *J. Geophys. Res. Solid Earth* **129**, e2023JB026941 (2024).

14. J. W. Hernlund, I. Bonati, Modeling ultralow velocity zones as a thin chemically distinct dense layer at the core-mantle boundary. *J. Geophys. Res. Solid Earth* **124**, 7902–7917 (2019).

15. J. Dannberg, R. Myhill, R. Gassmoller, S. Cottaar, The morphology, evolution and seismic visibility of partial melt at the core–mantle boundary: Implications for ULVZs. *Geophys. J. Int.* **227**, 1028–1059 (2021).

16. M. Li, A. K. McNamara, E. J. Garnero, S. Yu, Compositionally-distinct ultra-low velocity zones on Earth's core-mantle boundary. *Nat. Commun.* **8**, 177 (2017).

17. A. Mundl-Petermeier, R. J. Walker, M. G. Jackson, J. Blichert-Toft, M. D. Kurz, S. A. Halldórsson, Temporal evolution of primordial tungsten-182 and $^{3}He/^{4}He$ signatures in the Iceland mantle plume. *Chem. Geol.* **525**, 245–259 (2019).

18. A. Mundl-Petermeier, R. J. Walker, R. A. Fischer, V. Lekic, M. G. Jackson, M. D. Kurz, Anomalous ^{182}W in high $^{3}He/^{4}He$ ocean island basalts: Fingerprints of Earth's core? *Geochim. Cosmochim. Acta* **271**, 194–211 (2020).

19. A. L. Ferrick, J. Korenaga, Long-term core–mantle interaction explains W-He isotope heterogeneities. *Proc. Natl. Acad. Sci. U.S.A.* **120**, e2215903120 (2023).

20. M. Avants, T. Lay, E. J. Garnero, A new probe of ULVZ *S*-wave velocity structure: Array stacking of *ScS* waveforms. *Geophys. Res. Lett.* **33**, L07314 (2006).

21. J. Jenkins, S. Mousavi, Z. Li, S. Cottaar, A high-resolution map of Hawaiian ULVZ morphology from *ScS* phases. *Earth Planet. Sci. Lett.* **563**, 116885 (2021).

22. M. S. Thorne, S. Pachhai, K. Leng, J. K. Wicks, T. Nissen-Meyer, New candidate ultralow velocity zone locations from highly anomalous *SPdKS* waveforms. *Minerals* **10**, 211 (2020).

23. Y. Xu, K. D. Koper, Detection of a ULVZ at the base of the mantle beneath the northwest Pacific. *Geophys. Res. Lett.* **36**, L17301 (2009).

24. D. Kim, V. Lekic, A. Mundl-Petermeier, V. Finlayson, R. Walker, "Sequencing core diffracting seismic phases: Implications for mega-ULVZ properties," in *AGU Fall Meeting Abstracts* (American Geophysical Union, 2021), vol. 2021, pp. 13–17.

25. L. Jagt, C. Martin, F. Millet, S. Russell, S. Cottaar, P_{diff} postcursors from the base of the Hawaiian ULVZ. *Seismic Record* **4**, 204–213 (2024).

26. A. M. Dziewonski, D. L. Anderson, Preliminary reference Earth model. *Phys. Earth Planet Int.* **25**, 297–356 (1981).

27. M. van Driel, L. Krischer, S. C. Stahler, K. Hosseini, T. Nissen-Meyer, Instaseis: Instant global seismograms based on a broadband waveform database. *Solid Earth* **6**, 701–717 (2015).

28. D. Baron, B. Menard, Extracting the main trend in a data set: The sequencer algorithm. *Astrophys. J.* **916**, 91 (2021).

29. J. Wolf, E. Garnero, B. Schwarz, K. Leng, Y. Luo, R. Maass, J. D. West, Detection of lowermost mantle heterogeneity using seismic migration of diffracted *S*-waves. *J. Geophys. Res. Solid Earth* **130**, e2025JB031367 (2025).

30. C. Martin, T. Bodin, S. Cottaar, Mapping structures on the core–mantle boundary using S_{diff} postcursors: Part II. Application to the Hawaiian ULVZ. *Geophys. J. Int.* **235**, 2399–2409 (2023).

31. Z. Li, K. Leng, J. Jenkins, S. Cottaar, Kilometer-scale structure on the core–mantle boundary near Hawaii. *Nat. Commun.* **13**, 2787 (2022).

32. A. K. McNamara, E. J. Garnero, S. Rost, Tracking deep mantle reservoirs with ultra-low velocity zones. *Earth Planet. Sci. Lett.* **299**, 1–9 (2010).

33. T. Lay, J. Hernlund, E. J. Garnero, M. S. Thorne, A post-perovskite lens and D heat flux beneath the central Pacific. *Science* **314**, 1272–1276 (2006).

34. C. Zhao, E. J. Garnero, M. Li, A. McNamara, S. Yu, Intermittent and lateral varying ULVZ structure at the northeastern margin of the Pacific LLSVP. *J. Geophys. Res. Solid Earth* **122**, 1198–1220 (2017).

35. J. Ritsema, E. J. Garnero, T. Lay, A strongly negative shear velocity gradient and lateral variability in the lowermost mantle beneath the Pacific. *J. Geophys. Res. Solid Earth* **102**, 20395–20411 (1997).

36. J. K. Wicks, J. M. Jackson, W. Sturhahn, D. Zhang, Sound velocity and density of magnesiowüstites: Implications for ultralow-velocity zone topography. *Geophys. Res. Lett.* **44**, 2148–2158 (2017).

37. J. P. Watt, G. F. Davies, R. J. O'Connell, The elastic properties of composite materials. *Rev. Geophys.* **14**, 541–563 (1976).

38. V. H. Lai, D. V. Helmberger, V. V. Dobrosavljevic, W. Wu, D. Sun, J. M. Jackson, M. Gurnis, Strong ULVZ and slab interaction at the northeastern edge of the Pacific LLSVP favors plume generation. *Geochem. Geophys. Geosyst.* **23**, e2021GC010020 (2022).

39. K. Ohta, R. E. Cohen, K. Hirose, K. Haule, K. Shimizu, Y. Ohishi, Experimental and theoretical evidence for pressure-induced metallization in FeO with rocksalt-type structure. *Phys. Rev. Lett.* **108**, 026403 (2012).

40. W.-G. D. Ho, P. Zhang, K. Haule, J. M. Jackson, V. Dobrosavljević, V. V. Dobrosavljevic, Quantum critical phase of FeO spans conditions of Earth's lower mantle. *Nat. Commun.* **15**, 3461 (2024).

41. M. Manga, R. Jeanloz, Implications of a metal-bearing chemical boundary layer in D for mantle dynamics. *Geophys. Res. Lett.* **23**, 3091–3094 (1996).

42. Y. Su, S. Ni, B. Zhang, Y. Chen, W. Wu, M. Li, H. Sun, M. Hou, X. Cui, D. Sun, Detections of ultralow velocity zones in high-velocity lowermost mantle linked to subducted slabs. *Nat. Geosci.* **17**, 332–339 (2024).

43. M. M. Festin, M. S. Thorne, M. Li, Evidence for ultra-low velocity zone genesis in downwelling subducted slabs at the core–mantle boundary. *Seismic Record* **4**, 111–120 (2024).

44. S. E. Hansen, E. J. Garnero, M. Li, S.-H. Shim, S. Rost, Globally distributed subducted materials along the Earth's core-mantle boundary: Implications for ultralow velocity zones. *Sci. Adv.* **9**, eadd4838 (2023).

45. K. Kawano, M. Nishi, H. Kuwahara, S. Kakizawa, T. Inoue, T. Kondo, Extensive iron–water exchange at Earth's core–mantle boundary can explain seismic anomalies. *Nat. Commun.* **15**, 8701 (2024).

46. S. Pachhai, M. Li, M. S. Thorne, J. Dettmer, H. Tkalcic, Internal structure of ultralow-velocity zones consistent with origin from a basal magma ocean. *Nat. Geosci.* **15**, 79–84 (2022).

47. D. J. Bower, J. K. Wicks, M. Gurnis, J. M. Jackson, A geodynamic and mineral physics model of a solid-state ultralow-velocity zone. *Earth Planet. Sci. Lett.* **303**, 193–202 (2011).

48. S. Russell, J. C. Irving, L. Jagt, S. Cottaar, Evidence for a kilometer-scale seismically slow layer atop the core-mantle boundary from normal modes. *Geophys. Res. Lett.* **50**, e2023GL105684 (2023).

49. C.-E. Boukare, Y. Ricard, G. Fiquet, Thermodynamics of the MgO-FeO-SiO_2 system up to 140 GPa: Application to the crystallization of Earth's magma ocean. *J. Geophys. Res. Solid Earth* **120**, 6085–6101 (2015).

50. F. Nabiei, J. Badro, C. É. Boukaré, C. Hébert, M. Cantoni, S. Borensztajn, N. Wehr, P. Gillet, Investigating magma ocean solidification on earth through laser-heated diamond anvil cell experiments. *Geophys. Res. Lett.* **48**, e2021GL092446 (2021).

51. C. É. Boukaré, J. Badro, H. Samuel, Solidification of Earth's mantle led inevitably to a basal magma ocean. *Nature* **640**, 114–119 (2025).

52. H. Rizo, D. Andrault, N. R. Bennett, M. Humayun, A. Brandon, I. Vlastélic, B. Moine, A. Poirier, M. A. Bouhifd, D. T. Murphy, ^{182}W evidence for core-mantle interaction in the source of mantle plumes. *Geochem. Perspect. Lett.* **11**, 6–11 (2019).

53. A. M. Dziewonski, T.-A. Chou, J. H. Woodhouse, Determination of earthquake source parameters from waveform data for studies of global and regional seismicity. *J. Geophys. Res. Solid Earth* **86**, 2825–2852 (1981).

54. L. Van der Maaten, G. Hinton, Visualizing data using t-SNE. *J. Mach. Learn. Res.* **9**, 2579–2605 (2008).

55. J. Tromp, D. Komatitsch, Q. Liu, Spectral-element and adjoint methods in seismology. *Commun. Comput. Phys.* **3**, 1–32 (2008).

56. K. Leng, T. Nissen-Meyer, M. Van Driel, K. Hosseini, D. Al-Attar, AxiSEM3D: Broad-band seismic wavefields in 3-D global earth models with undulating discontinuities. *Geophys. J. Int.* **217**, 2125–2146 (2019).

57. E. Vanacore, S. Rost, M. Thorne, Ultralow-velocity zone geometries resolved by multidimensional waveform modelling. *Geophys. J. Int.* **206**, 659–674 (2016).

58. V. V. Dobrosavljevic, W. Sturhahn, J. M. Jackson, Evaluating the role of iron-rich (Mg,Fe)O in ultralow velocity zones. *Minerals* **9**, 762 (2019).

59. S. M. Dorfman, F. Nabiei, C. E. Boukaré, V. B. Prakapenka, M. Cantoni, J. Badro, P. Gillet, Composition and pressure effects on partitioning of ferrous iron in iron-rich lower mantle heterogeneities. *Minerals* **11**, 512 (2021).

60. Y. Tange, E. Takahashi, Y. Nishihara, K.-i. Funakoshi, N. Sata, Phase relations in the system $MgO-FeO-SiO_2$ to 50 GPa and 2000°C: An application of experimental techniques using multianvil apparatus with sintered diamond anvils. *J. Geophys. Res. Solid Earth* **114**, B02214 (2009).

61. Y. Takei, Effect of pore geometry on V/V: From equilibrium geometry to crack. *J. Geophys. Res. Solid Earth* **107**, ECV 6–1–ECV 6–12 (2002).

62. C. Y. Shi, L. Zhang, W. Yang, Y. Liu, J. Wang, Y. Meng, J. C. Andrews, W. L. Mao, Formation of an interconnected network of iron melt at Earth's lower mantle conditions. *Nat. Geosci.* **6**, 971–975 (2013).

63. T. Komabayashi, Thermodynamics of melting relations in the system Fe-FeO at high pressure: Implications for oxygen in the Earth's core. *J. Geophys. Res. Solid. Earth* **119**, 4164–4177 (2014).

64. W. L. Mao, H. Mao, W. Sturhahn, J. Zhao, V. B. Prakapenka, Y. Meng, J. Shu, Y. Fei, R. J. Hemley, Iron-rich post-perovskite and the origin of ultralow-velocity zones. *Science* **312**, 564–565 (2006).

65. R. Caracas, R. E. Cohen, Effect of chemistry on the stability and elasticity of the perovskite and post-perovskite phases in the $MgSiO_3-FeSiO_3-Al_2O_3$ system and implications for the lowermost mantle. *Geophys. Res. Lett.* **32**, L16310 (2005).

66. T. Liu, X. Wang, C. Pu, Z. Jing, Thermoelastic properties of seifertite at high pressures and temperatures: Implications for negative velocity discontinuities in the D” layer. *Geophys. Res. Lett.* **51**, e2024GL112270 (2024).

67. W. Wang, Y. Xu, D. Sun, S. Ni, R. Wentzcovitch, Z. Wu, Velocity and density characteristics of subducted oceanic crust and the origin of lower-mantle heterogeneities. *Nat. Commun.* **11**, 64 (2020).
